# Human and the beast—Flight and aggressive responses of European bison to human disturbance

**DOI:** 10.1371/journal.pone.0200635

**Published:** 2018-08-01

**Authors:** Andżelika Haidt, Tomasz Kamiński, Tomasz Borowik, Rafał Kowalczyk

**Affiliations:** 1 Forest Research Institute, Sękocin Stary, Raszyn, Poland; 2 Mammal Research Institute, Polish Academy of Sciences, Białowieża, Poland; Universita degli Studi di Sassari, ITALY

## Abstract

Large mammals are often a source of conflict, and consequently there has been increasing interest in close encounters with them. Knowledge of wildlife responses to human disturbance is crucial for the management of increasing and expanding populations of large animals. We investigated flight initiation distance (FID) and aggressive responses of the European bison–the largest terrestrial mammal of Europe–to human disturbance in the Białowieża Forest (NE Poland). When encountered by humans, bison usually flee. Aggression was observed in only 0.4% of approach attempts. Mean FID was 77±46 m and was influenced by habitat, sex, and supplementary feeding intensity. Females showed greater timidity than males, FID was lower in forest than in open habitats, and supplementary feeding caused a drop in FID. In 84.5% of all documented aggression cases, bison attacks were provoked by humans approaching too close to the bison or by deliberate scaring them. Males were more aggressive than females, and attacked mainly during the rut, while females attacked during the winter and calving. Bison attacked in built-up areas significantly more often than expected. The mean critical distance of attacks was 21±2 m. Most attacks took the form of a short chase preceded by warning signs. Goring was observed in 22.7% of all aggression cases and no fatalities were recorded. Our study shows that bison are not dangerous animals and only manifest aggression in response to prolonged disturbance at close ranges. The education of people and recommendations for minimum approach distances should ensure a low rate of disturbance and safety when encountering large mammals.

## Introduction

Large mammals can be a source of conflict, instigating crop depredation, the killing of livestock, human injury, and even fatality in the most severe cases [1,2,3]. Many such conflicts occur in Africa and southern Asia, where the majority of large mammals are distributed and increasingly overlap with human-occupied areas [2,4,5]. In Europe, the rate of conflict is generally low; however, damage to farm crops by large ungulates, livestock depredation, or even attacks by large carnivores on people do occur [6,7,8]. Although densely inhabited by humans and with strongly fragmented habitats, Europe is inhabited by an increasing population of large mammals [9,10]. This suggests that both disturbances to wildlife and the frequency of encounters between humans and large animals will increase. On the other hand, ecotourism and outdoor activities are expanding all over the world, but the effects of these activities are not entirely benign to wildlife [11,12]. Large mammals, especially rare and charismatic species, are great tourist attractions and contribute both directly and indirectly to local and national economies through revenue generation [13]. The increasing interest of close encounters with wildlife and a lack of persecution often leads to decreases in animal fear, which may increase risk to humans [14,15]. People often break certain rules in their quests to observe large mammals. On the other hand, the fear of large mammals can be a major obstacle to their reintroductions [16], even though attacks on people are rare and often caused by human imprudence [14].

In prey-species, behavioral strategies are based on the trade-off between the need for acquiring food and the need for safety [17]. Despite lethal (consumptive) effects, predation may have non-lethal, trait-mediated effects on prey communities [18]. Non-lethal effects are associated with decreased fitness through limited access to preferred habitats, disrupted social structure, increased stress, and energy expenditures [19]. Thus, prey-species usually avoid confrontation with predators, including humans [20], though their decisions are achieved by weighing the costs and benefits of alternative responses [21]. After a safe distance is crossed, they usually flee [22,23], and in rare cases their response is one of defense and attack [24]. The distance at which animals begin to flee is referred to as the flight-initiation distance (FID) [25]. This differs by species, and can be influenced by many variables such as sex, age or reproductive status of animals, group size, environmental factors, season, intruder starting distance, and rate of disturbance [26,27,28]. Frequent contact between animals and humans resulting from the repeated exposure of animals to human observations or management actions, such as supplementary feeding, may lead to increased tolerance to humans and drop of FID [29,30]. This may especially apply to ‘refugee species’, such as European bison *Bison bonasus*, which have been restored in marginal habitats and require supplementary feeding in the winter [31].

The European bison is the largest terrestrial mammal of Europe: the body mass of an adult male can reach 840 kg [32]. After extinction in the wild at the beginning of 20^th^ century, they were restored from captive survivors and re-introduced to 35 locations in Europe [33]. The number of bison is steadily increasing and there were nearly 4,500 of them in the wild in 2016 [34]. Bison are adapted to open and mixed habitats [31,35]. Introduced mainly to forest habitats, they increasingly spread to farmlands and the vicinities of human settlements, which now increases the risk and rate of conflicts [36]. To reduce bison migration to farmlands and mitigate damage to farm crops in winter, bison are supplementarily fed at fixed locations. Bison are quite rarely killed by large predators and the main cause of their mortality is sanitary and selective culling [33]. The largest bison population, numbering over 1,100 individuals, inhabits the Białowieża Forest [34]. Bison have for a long time been recognized as the flagship species in the area, driving local development based on wildlife resources and attracting increasing numbers of tourists. This has especially been observed in the last 25 years, since democratic changes in Poland and improvement of the economic situation of citizens [37,38] has stimulated increasing interest in ecotourism.

Greater interest in re-introducing bison to other areas, including the more densely inhabited Western and Southern Europe, requires knowledge of bison behaviour in response to human disturbance. In 2013–2014, the first bison were released into the wild in western Germany and Romania, with further releases being planned in other countries. Reintroduction often raises questions about the potential risk of conflict relating to the presence of this large herbivore and the safety of residents and tourists.

The main aim of this study was to investigate the behavioural response of European bison to human disturbance and evaluate flight initiation and critical distance for this large herbivore. We asked: 1) what factors influence bison response to human encounters, and 2) how do management actions (i.e. winter supplementary feeding) influence bison behaviour. We predicted that female bison would show greater timidity than males, and that supplementary feeding leads to increased habituation of bison and a drop of FID.

Finally, based on documented cases of aggressive bison behaviour towards humans, we examined the reasons and nature of bison attacks. The results of this study are important for the conservation management of bison and other large herbivores; they may help guide future reintroduction programmes and develop set-back distances (i.e., the distances between humans and bison that result in no appreciable disturbance of this large herbivore) in existing and planned populations, in addition to helping manage human-wildlife conflicts, reduce human disturbance, and ensure the safety of people.

## Materials and methods

### Study area

The study was carried out in the Polish part of the Białowieża Forest (BF) (52°29'–52°37'N, 23°31'–24°21'E)—the best preserved forest in lowland Europe—located in NE Poland on the border with Belarus. The Polish part of the BF covers 600 km^2^, 16% of which is protected as the Białowieża National Park. Within the BF, 94% of the area constitutes deciduous and mixed continuous forest, and 6% is open habitat which includes forest glades, river valleys, and human settlements [39]. The region is characterized by low human density (30 people/km^2^). The majority of human settlements are located on the edges and outside of the forest, with very few villages located within the forest. Annually, over 150,000 tourists visit the BF, mainly during the spring and summer seasons.

The climate of BF is transitional between Atlantic and continental types with clearly marked seasons. The mean annual temperature is 7°C. The coldest month is January with an average temperature of -4.8°C, while the warmest is July with an average of 18.4°C. Snow cover persists for 60–96 days a year with a maximum recorded depth of 95 cm [40].

The bison population in the Polish part of BF numbers 590 individuals [34] and utilizes an area of over 800 km^2^ which includes BF and its surroundings [36,41]. Males roam solitarily (60% of individuals) or in small groups (up to 8 individuals), females create herds of 10–15 individuals on average consisting of cows, calves, and subadults [33]. Calving takes place mainly between May and July, and the rutting season begins in mid-July and ends in October [33,42]. In the winter, bison are supplementarily fed at 6–7 feeding sites with differing frequency or migrate to agricultural areas and feed on a variety of resources, including hay left by farmers in meadows [41,43].

### Data collection

For FID analysis, experimental human approaches towards free-living bison were conducted from October 2009 to February 2013 during different activities related to bison research including bison survey, radio-tracking and radio-collaring. Bison were located during field surveys and through radio-tracking. All approaches were conducted by two experienced observers at standard walking speed. When possible, some approaches were conducted by car to check the influence of vehicles on bison behaviour. During the approach, the distance to the animals was measured using a laser rangefinder (Bushnell) with 5 m accuracy. All cases of aggressive behaviour of bison were recorded. A total of 465 approaches were conducted (S1 Table).

We estimated (1) alert distance (AD)–the distance between the bison and the observer at which the vigilance response was initiated (bison raised its head and looked towards the approaching observer); (2) flight initiation distance (FID)–the distance between the bison and the observer at the moment when the bison started to move away. Other recorded data were as follows: 1) the habitat (closed forest) or open); 2) bison sex; 3) herd size; 4) approach type (on foot or by car). Additional variables used in the analysis were the intensity of supplementary feeding in winter (intensively fed–food delivered to fixed locations 3–5 times a week; less intensively fed–food delivered 1–2 times a week; and non fed bison), season (winter (Dec-Mar) or spring-autumn (Apr-Nov)), and hour.

Data on aggressive bison behaviour were documented through direct interviews with victims or witnesses of bison attacks. The survey included the following information: 1) place and date of the aggressive bison behaviour; 2) reasons for the attack; 3) habitat (forest, open, built-up area); 4) bison sex; 5) critical distance–estimated distance between human and bison at the moment of the attack; 6) duration of bison disturbance before the attack; 7) form of attack (gore or chase); 8) occupation of the attacked person; 9) forms of warning by the bison before the attack. In total between 1979 and 2015, 45 cases of aggressive bison behaviour towards humans were reported, including those recorded during approaches for FID data collection.

Within the area occupied by bison in BF, the proportion of main habitats used in the analysis of attack distribution was estimated using Arc GIS [44].

### Ethical statement

This study was carried out under research permits no. DLOPiK-op/ogiz-4200/IV.A-38-1/8310,10568/07/wo from the Polish Ministry of Environment and no. DOPozgiz-4200/IV.A-4/208/10/ls from the General Director for Environmental Protection in Poland, as well as ethics permits no. 31/2006 and 2009/52 from the Local Ethical Commission in Białystok, Poland.

All interviewed persons gave verbal consent for the use of provided data on bison aggressive behaviour. No minors were interviewed.

### Statistical analysis

In order to investigate the effects of predictor variables–the hour of observation, approach type, bison sex, herd size, season, and habitat–on the FID of bison, we fitted generalized linear models (GLMs). As the response variable had a non-normal distribution, we applied GLM with a gamma-distributed dependent variable (with log link function). The explanatory variables were weakly correlated (*r* < |0.24|), with the exception of herd size and sex (r = 0.62). Thus all the variables, except herd size, were included in the modelling procedure. The global model contained the main effects of all explanatory variables as well as the interaction between habitat and season. In addition, we investigated the influence of supplementary winter feeding on FID. We achieved this by applying gamma GLM exclusively to winter data. When modelling the winter data set, we used the same explanatory variables as above but replaced season with feeding intensity and excluded habitat as it was significantly correlated with feeding intensity (r = 0.48, *P* < 0.001). Feeding intensity was expressed at three levels (see Data collection). The global model for winter data included the main effects of all explanatory variables: the hour of observation, approach type, bison sex, and feeding intensity. No interactions were included in winter models.

To rank models we used the Akaike Information Criterion (AIC) with a second-order correction for the small sample size (AICc; [45]). Since we did not find a singular best model for neither the first set of models (total data) nor the second (winter data), we applied model averaging. We averaged over the subsets of models whose cumulative weights did not exceed 0.95. We checked the homoscedasticity of model residuals by inspection of the model residual plot projected against fitted values (estimated responses). All statistical analyses were made with R (version 3.1.2; R Development Core Team 2014).

## Results

### Flight initiation distance

**Fig 1:**
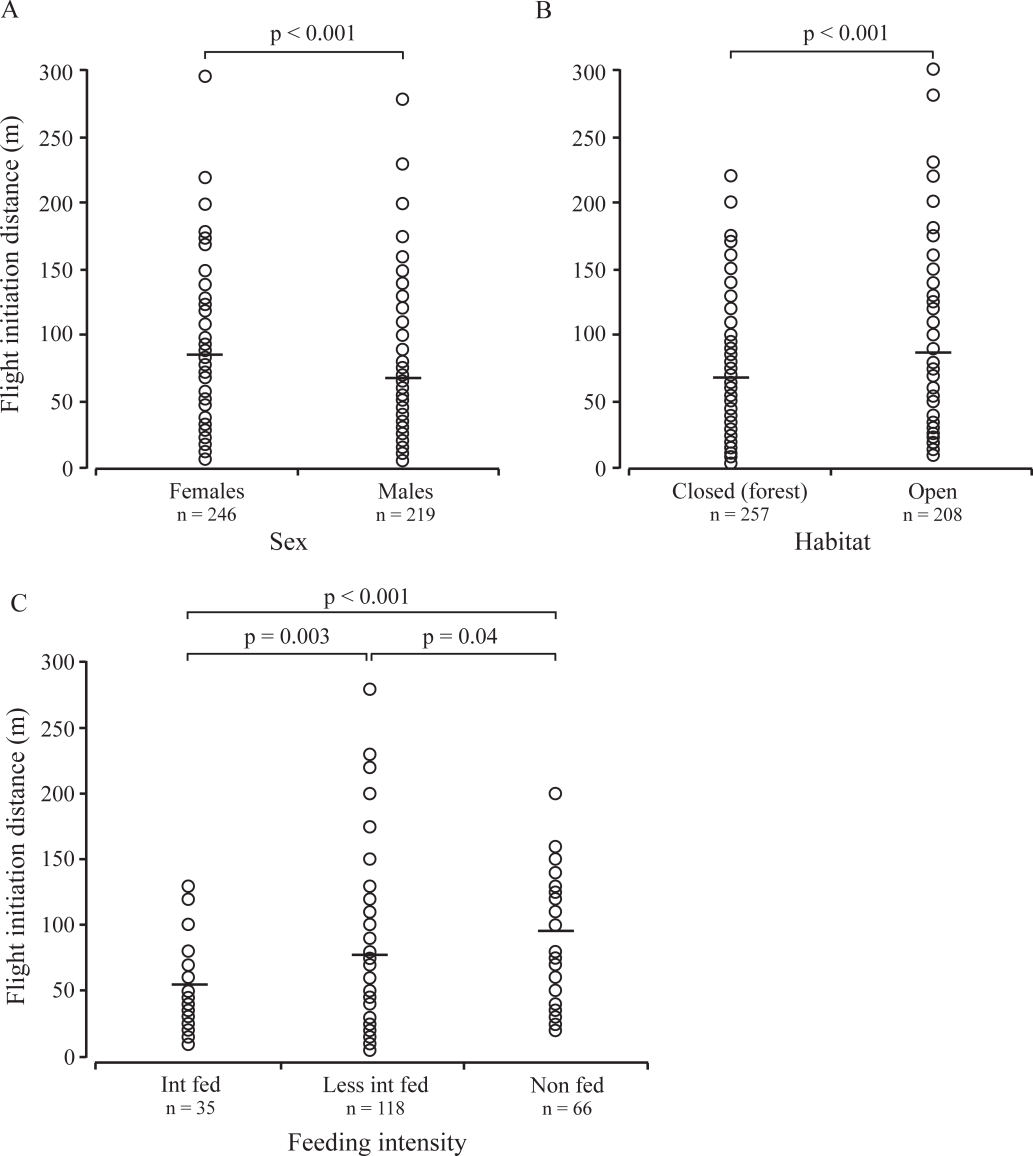
Influence of sex (A), habitat (B), and supplementary feeding intensity (C) on FID of European bison in Białowieża Forest.

The mean FID (±SD) of European bison was 77±46 m, (range: 5–300 m, *N* = 465), and average AD was 110±62 m (range: 10–350 m). For all data, analysis of the factors affecting FID in bison indicated the significant effects of two explanatory factors–habitat and sex (Table 1). The FID of bison was significantly higher in females than in males (86±46 and 68±46 m, respectively), and lower in closed (forest) than in open habitats (68±39 and 89±51 m, respectively) (Table 2; Fig 1A–1B).

**Table 1:** Model selection (based on the AICc criteria) for the considered gamma generalized linear models for data covering both the whole year and winter (December-March) exclusively. The models aim to assess the effects of different factors* on the flight initiation distance of individuals in the bison population in Białowieża Forest (only models whose cumulative weights (ω*i*) were below 0.95 and intercept models are presented). For both datasets (whole year and winter data) model averaging (cumulative weights ≤ 0.95) was applied.

Model	*K*	*R*^*2*^	AIC_c_	ΔAIC_c_	ω*i*
*Whole year*					
Hab + AT + Sex	5	0.10	4716.5	0	0.251
Hab + Sex	4	0.09	4717.3	0.82	0.166
Hab + AT + S + Sex	6	0.10	4717.8	1.31	0.130
Hab + Hour + AT + Sex	6	0.10	4718.4	1.89	0.098
Hab + S + Sex	5	0.09	4719.0	2.54	0.071
Hab + AT + S + Sex + Hab×S	7	0.10	4719.1	2.63	0.068
Hab + Hour + Sex	5	0.09	4719.2	2.69	0.065
Hab + Hour + AT + S + Sex	7	0.10	4719.7	3.22	0.050
Hab + S + Sex + Hab×S	6	0.09	4720.5	3.99	0.034
Intercept	2	0	4757.3	40.78	0
*Winter*					
Suppl_feed	4	0.08	2241.2	0	0.243
Suppl_feed + Sex	5	0.08	2241.8	0.62	0.178
Suppl_feed + Hour	5	0.08	2241.9	0.71	0.171
Suppl_feed + Hour + Sex	6	0.09	2242.6	1.43	0.119
Suppl_feed + AT	5	0.08	2242.9	1.72	0.103
Suppl_feed + AT + Sex	6	0.09	2243.7	2.46	0.071
Intercept	2	0	2252.2	13.96	0

*Hab–habitat (open, closed); Hour; AT–approach type (on foot, by car); S–season (non-winter, winter); Sex (male, female); Suppl_feed–supplementary feeding (intensively fed, less intensively fed, non-fed); *K–*number of estimated parameters; AIC_c_−Akaike’s information criterion with a second order correction for small sample sizes; ΔAICc−difference in AIC_c_ between a given model and the most parsimonious model; ω*i*–weight of the model. Parameter estimates of the averaged models (whole year data and winter data) are presented in Table 2.

**Table 2:** Averaged parameter estimates for the gamma generalized linear models of whole year data and winter data (Table 1), which describe the effects of different factors* on the flight initiation distance of individuals in the bison population in Białowieża Forest (2009–2013).

Variables*	Estimate	SE	*z* value	*P* value
*Whole year*				
Intercept	4.34	0.09	47.89	<0.001
Habitat open (closed)	0.27	0.06	4.42	<0.001
Hour	0.004	0.01	0.39	0.70
Approach type on foot (by car)	-0.10	0.06	1.68	0.09
Season non winter (winter)	0.05	0.06	0.83	0.41
Sex male (female)	-0.24	0.05	4.43	<0.001
Habitat open (closed) × Season non winter (winter)	-0.09	0.11	0.81	0.42
*Winter*				
Intercept	4.10	0.19	21.60	<0.001
Feeding intensity				
less_int_fed (int_fed)	0.34	0.12	2.94	0.003
non_fed (int_fed)	0.54	0.12	4.32	<0.001
less_int_fed (non_fed)	-0.19	0.09	2.07	0.04
Hour	-0.02	0.02	1.06	0.29
Approach type on foot (by car)	-0.05	0.08	0.57	0.57
Sex male (female)	-0.10	0.08	1.06	0.26

Reference levels for analysed factors are presented in parenthesis.

For winter data, the average model showed a significant effect of supplementary winter feeding intensity on FID in bison (Table 2). Intensively fed animals were characterised by significantly shorter FID (55±34 m) compared to less intensively and non-fed bison (77±48 and 94±45 m, respectively); less intensively fed bison had significantly lower FID than non-fed bison (Table 2, Fig 1C).

Approach type, season, and hour of day did not affect the FID for any of the considered models (Table 2).

### Bison aggressive behaviour

**Fig 2:**
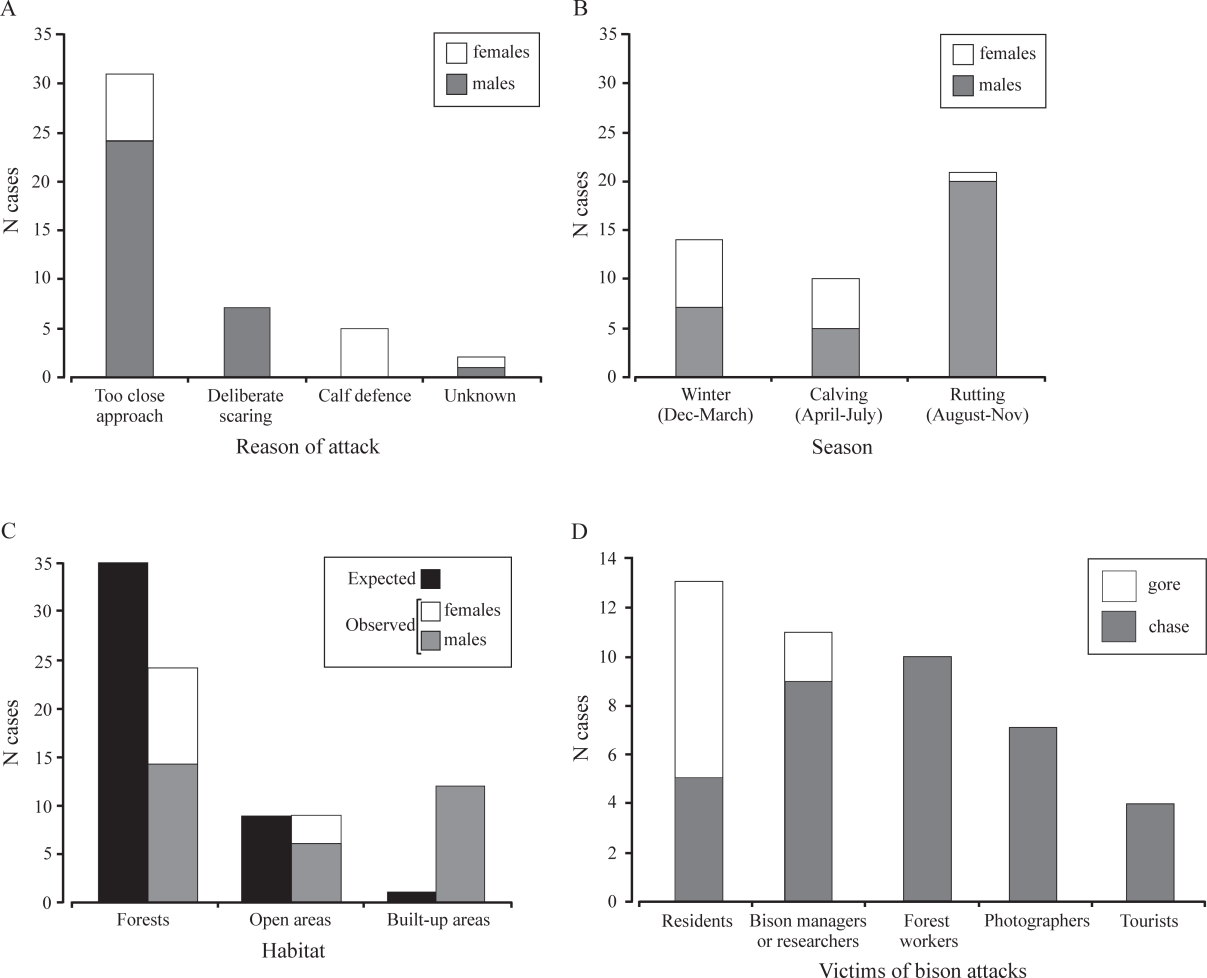
Reasons (A), seasonal distribution (B), habitat distribution (C), and victims (D) of European bison aggressive responses in Białowieża Forest.

During FID estimation in 2009–2012, two cases of bison attack were recorded upon approach by an observer (i.e. 0.4% of cases). Analysis of all documented cases of bison aggressive behaviour (*N* = 45) determined that 1.2 aggressive responses were recorded per year. Bulls showed this behaviour more often (71.1% of cases) than cows (28.9%). In all cases, attacks were by single individuals; however, in 31.1% of cases the aggressive bison was accompanied by other individuals (from 2 to 120). The main reason for bison aggressive responses was due to too close of an approach to the individual (68.9%); followed by deliberate scaring (15.6%), which was observed only in males; and calf defence (11.1%), observed only in females (Fig 2A). Males and females significantly differed in reasons for aggression (G-test for homogeneity, *G* = 76.7, *P* < 0.001) and seasonal distribution of attacks (G-test, *G* = 72.6, *P* < 0.001). Males attacked mainly during the rut, while females during winter and calving (Fig 2B).

Most of the attacks took place in forest habitats (53.3%), followed by built-up areas (26.7%) and open areas (20.0%). Taking into account the proportion of different habitats available, the attacks in forests occurred significantly less frequently than expected, while attacks in built-up areas were significantly more frequent than expected (G-test, *G* = 30.3, *P* < 0.001) (Fig 2C). All attacks in built-up areas were by males.

The average critical distance of attack (mean ± SE) was 21±2 m (range: 2–50 m), and did not differ between males and females (Mann-Whitney test, *U* = 186.5, *P* > 0.4). Bison were observed before attack in 88.1% of cases, for an average time of 14 ± 2 minutes (range: 3–60 min). In 11.9% cases, bison were first noticed at the moment of attack.

Bison warned their targets about the expected attack in 44.1% of cases. Warning was significantly more often observed in males than females (54.8% and 15.4% cases of attacks, respectively) (G-test, *G* = 35.6, *P* < 0.001). The most frequent way of warning was head swinging (24.4% cases) and hoofing the ground (22.2%) (Table 3). In 22.2% of cases, bison manifested warning in two or more ways.

**Table 3:** Types of signalling by European bison when warning before attack.

The type of signal when warning	(%)
No warning	55.9
Head swinging	24.4
Hoofing the ground	22.2
Tail flicking	13.3
Grunting	8.9
Wallowing, tree rubbing or damaging	6.6
Approaching	2.2

In 40.9% of cases, bison exhibited aggression towards people that were involved with the bison (managers, researchers, and photographers) (Fig 2D). Residents and forest workers were the victims of attack in 27.3% and 22.7% of cases, respectively. Rarely was bison aggression directed at tourists (Fig 2D).

The most frequent form of aggressive behaviour was a short chase (77.3% of cases). A gore was observed in 10 cases (22.7% of attacks), i.e. 0.3 gore attacks per year, always by males. In eight cases the victims of gore attacks were residents, while in two they were people in some way involved with the bison (Fig 2D). In eight cases gore ended with injuries. No fatal cases were recorded.

## Discussion

We found bison FID to be among the lowest observed in ungulates [46,47,48,49,50,51,52]. This may be related to the relatively low risk of predation, as European bison are rarely predated upon by wolves [53], but have frequent close encounters with humans. Bison are subject to limited selective culling in BF [31], and seasonally higher tourist pressure occurs. Thus, humans are the main predator for bison and their responses are adjusted mainly to disturbances induced by them. However, disturbance is also related to food provision. When animals are repeatedly exposed to close encounters with humans without negative consequences, they conserve energy by muting their responses [30]. This leads to habituation and closer distance tolerance towards observers or predators. In the BF, supplementary feeding is most likely the main factor responsible for bison having a less distinct fear of humans. Supplementary feeding is a common management practice in bison populations [31], and may lead not only to habituation, but also to attraction by strengthening an animal’s behaviour through positive reinforcement and encouraging movement towards a stimuli [54]. The presence of humans is thus associated with the delivery of food, thereby the fear barrier and the reaction distance of the animal reduces, despite occasional culling. This is especially observed in intensively fed herds in BF, which stay for up to four months at feeding sites served by rangers with hay (occasionally also beetroots) 3–5 times a week [55]. Decreased FID could in fact be the result of differences in the availability of foraging habitats elsewhere [56]. Bison may not avoid disturbance because they have little access to alternative feeding sites and are thus constrained to stay in disturbed areas. Reintroduced mainly to forest habitats, where forage for large herbivores is strongly limited in the winter [31], bison aggregate at a few feeding sites or utilize meadows (covering 3% of the area) and farmland out of the forest [8,36]. Animals with limited or no suitable habitat within close vicinity will be forced to stay despite disturbance [56]. Thus during the winter in BF, food patch availability is limited for bison, especially in forests; this leads to increased habituation of bison to humans due to the provision of supplementary fodder or the maximization of fitness when costs of flight are higher than the benefits of staying on a good quality patch. This effect may increase in late winter especially, when bison body condition worsens and individuals ultimately select forage resources based on their higher nutrient content [41]. This reflects a trade-off whereby they accept a higher risk of human disturbance when starvation risk increases. Bison utilising agriculture areas had higher food patch availability [41], which resulted in higher FID, as the cost of flight was lower than in bison staying in the forest and remaining confined to winter feeding sites. An effect of supplementary feeding on bison behaviour has also been observed for resting site selection [57].

Female bison were more timid and responded at greater distances than males. This was probably due to the protection of their offspring as predicted by the reproductive strategy-predation risk hypothesis [58]. Females or groups with young offspring show greater flight responses than adult groups [28]. Bison females usually roam in mixed groups with calves and subadults and should minimize predation risk as their reproductive success is determined by survival of their offspring. Thus, females should display more cautious behaviour than males to ensure survival of their young and be more likely to react to predation risk and flight at greater distances [59]. Such contrasting behaviour of the two sexes has also been observed in other ungulates [48,50]. In mountain sheep *Ovis Canadensis*, for instance, higher levels of caution in females and their sensitive response to flee when encountered with coyotes resulted in lower mortality [60].

Lower FID of bison in forests in comparison to open habitat may be related to lower visibility in closed habitats and therefore limited ability of threat detection at longer distances [61]. Animals may flee as soon as they detect a threat. Thus, it is expected that in open habitats, the response to disturbance occurs earlier, which results in higher FID, as observed in bison in BF.

Aggression of bison was observed rarely. When encountered by humans, European bison usually flee, as shown by our study. We recorded 1.2 aggressive bison responses per year and 0.3 cases ending with injuries annually. By comparison, American bison in Yellowstone National Park (YNP) were recorded with 4.8 aggression cases and 3.7 bison-caused injuries annually, including two fatalities [62,63]. Taking both the number of bison (600 in BF and 3,000 in YNP) and number of visitors per area unit (250 in BF and 330/km^2^ in YNP) into account, the close encounters between bison and humans in both YNP and BF are comparable.

In built-up areas in BF, bison showed aggression more often than expected. Wild animals are often attracted close to human settlements. Several hypotheses were considered with regards to this [64]. For bison, the most plausible is the food-conditioning hypothesis. Given the lack of winter forage within the forest, mowed meadows in the vicinity of human settlements are the most important foraging habitat for bison from autumn until early spring. Orchards with apple trees and vegetable gardens are also very attractive food resources. In combination with human-habituation (related to supplementary feeding), this increases the use of such areas, mainly by bison males, and the risk of human-bison conflicts. Comparatively, most of the aggressive behaviour of American bison took place in developed areas and along roads [63].

More cases of aggression were recorded during the rutting period, and these were almost exclusively by males. Aggressive interactions among males strongly increase during the rut [65,66]. This aggression can be taken out on humans that disturb bison during this period. This may especially apply to older bison, which often roam near human settlements and in open areas searching for attractive food resources [33]. When aging, they become more solitary and may show increasing aggressive behaviour [65]. If not expelled from nearby human settlements, they become insistent, increasing the risk of conflict.

Females attack humans while protecting their offspring [67,68]. In BF, according to study respondents, 38% of female aggression was due to calf defence, usually when the victims of attacks accidentally found themselves close to a calf. The rest of the aggressive responses of females were related to too close of an approach; however, some of them were probably also calf defensive behaviours, not detected by attacked persons. Aggressive defence of dependent offspring is a form of parental investment [69]. It may be directed towards intruders directly threatening young or towards intruders entering territories where young are being raised [69,70]. Seasonal habituation and contact of females with experienced rangers at feeding sites doesn’t lead to increased aggressive interactions. This is probably related to habituation and attraction, as well as food provision. Due to greater timidity, female close contacts with humans other than rangers are rare, while males that visit open areas or the vicinity of built-up ones frequently face encounters with residents, tourists, or nature photographers. This results in a much higher number of aggression cases towards these groups. While different groups of people were subject to bison aggression, gore and injuries were almost exclusively recorded among residents. This is probably due to their limited knowledge of the threat bison can pose, which resulted in low awareness and even direct aggression of the humans towards bison.

The majority of wild animal attacks are provoked by humans, usually because of unwelcome close and quick proximity [63,71]. Studies have shown that for carnivores, about half of well-documented reported attacks have involved risk-enhancing human behaviours [72]. This was also the case in this study, where 88% of attacks were provoked by humans. Relatively prolonged periods after which the bison attacks were triggered (14 minutes) and short critical distance of attacks (21 meters, on average) indicate that bison are provoked to attack when disturbed for a longer period of time and when approached very closely. All recorded cases of deliberate scaring of bison were by residents, and those people were usually victims of goring. Aggression towards people previously involved with bison rarely ended with goring, even though these people often cross safe distances due to their reduced fear of these large herbivores. This may result from their awareness of the threat posed, their calm behaviour in close proximity to bison, and the attention paid to the behavioural warning signs of bison. In almost half of the bison attacks, the victims noticed classic warning signs indicating agitation of bison. Reading these signals correctly would likely better protect victims from attack. Similarly to European bison, warning signs that an individual was about to gore were also observed in American bison [63]. Tourists were rarely victims of bison attacks; however, this value might be underestimated due to the limited access to information about such cases.

Conservation management should aim to reduce the effects of people on wildlife, while still allowing recreationists and ecotourists to view and photograph wildlife [73]. Using FID, wildlife managers try to develop set-back distances—the minimum distance that a human may approach without disturbing animals—to reduce the rates of human disturbance or avoid aggression from large animals [74,75]. Thus, set-back distances can be an effective conservation tool to reduce impacts on wildlife if they are carefully designed and derived from empirical evidence [76]. In most cases (90%), bison in BF fled when approached ≤ 130 m. Based on the 95% percentile of FID, as recommended by researchers [77], the set-back distance that reduces bison disturbance is ≥ 82 m. When taking into account only non-fed bison (FID = 94 m), it is ≥ 105 m. In relation to the level of bison management (intensively versus less intensively managed), these distances can be used as set-back distances to reduce human disturbance to bison.

## Conclusions

We conclude that bison are not aggressive animals and usually flee when encountered by humans. Management actions, namely the frequency of supplementary feeding, strongly influence the behaviour of bison and increase their habituation. Bison attack only in specific conditions when approached at a close distance and disturbed for a prolonged period of time. Most bison attacks have the form of a short chase and are directed mainly at people which cross this critical distance. One of the factors that increases conflict and the risk of aggressive behavioural interactions is the reintroduction of bison to non-optimal forest habitats. This leads to population expansion outside forest areas and selection for open habitats, and in effect increased encounter with humans.

The growing global population of bison (5% annually over the last decade), increasing interest of close encounters with wildlife, and the role that bison play in rewilding programmes [78] increase the importance of knowledge of their responses to human disturbance and potential human-wildlife conflicts. Humans can minimize their impact on large mammals, especially rare and endangered species, by using set-back distances and information on animal responses based on sound evidence. Education of local communities and tourists, as well as modification of management actions (less frequent feeding) may help mitigate bison disturbance and the risk of potential conflicts, and ultimately increase safety of people when encountering large animals.

## Supporting information

S1 Table (PDF)
