# Supplementary material for: Human and the beast—Flight and aggressive responses of European bison to human disturbance
Source: PLoS One. 2018 Aug 1;13(8):e0200635. doi: 10.1371/journal.pone.0200635 (PMC6070204; doi:10.1371/journal.pone.0200635)
Supplement: S1 Table — (PDF) [file pone.0200635.s001.pdf]

ID	Data	Hour	Season	Location	Feed	Hab.	Sex	Lokomo	Alert	FID
1	2009-10-12	13:00	2	242c	2	1	m	foot	50	50
2	2009-10-19	14:45	2	395b	1	1	m	foot	150	50
5	2009-10-25	12:00	2	397a	1	1	m	foot	125	75
6	2009-10-26	14:00	2	sieczniki M	2	2	m	foot	50	25
6	2009-10-26	13:45	2	sieczniki M	2	2	m	foot	100	50
7	2009-10-27	13:00	2	178a	2	1	m	foot	25	25
10	2009-10-30	12:00	2	629b/630a	2	1	f	foot	200	100
15	2009-11-05	14:15	2	445d	1	2	m	auto	150	50
16	2009-11-10	12:45	2	Zabrody	3	2	m	auto	100	25
17	2009-11-12	14:45	2	470d	1	2	m	auto	200	100
18	2009-11-12	14:30	2	470d	1	2	m	auto	75	50
19	2009-11-12	12:30	2	210b	2	1	f	foot	50	25
20	2009-11-12	11:30	2	335c	1	2	f	foot	100	50
21	2009-11-17	10:00	2	247d	1	1	f	foot	75	50
22	2009-11-20	12:45	2	421a	1	1	f	foot	100	50
26	2009-11-24	11:00	2	697a	2	1	m	auto	50	25
27	2009-11-25	7:45	2	Białowieża	2	2	m	auto	25	25
30	2009-12-07	9:00	1	697a	2	1	m	foot	5	5
33	2009-12-14	11:15	1	echy Orlańs	2	1	m	foot	50	25
34	2009-12-15	10:15	1	475c	2	1	m	foot	75	50
35	2009-12-21	13:15	1	echy Orlańs	2	1	m	foot	50	25
36	2009-12-21	12:45	1	697a	2	1	f	auto	75	75
38	2009-12-21	10:30	1	475a	2	2	m	auto	150	50
39	2009-12-22	13:00	1	sieczniki M	2	2	m	auto	200	50
40	2009-12-22	10:00	1	475a	2	2	m	auto	150	50
44	2010-01-04	14:30	1	Planta	3	2	m	auto	100	100
45	2010-01-04	14:00	1	Zabrody	3	2	f	foot	100	50
46	2010-01-04	13:30	1	Zabrody	3	2	f	auto	200	100
48	2010-01-05	13:30	1	Biała Straż	3	2	m	foot	200	200
49	2010-01-05	13:45	1	sieczniki M	2	2	m	auto	25	25
50	2010-01-05	12:45	1	Krugłe	3	2	m	auto	75	75
51	2010-01-05	10:45	1	697a	2	1	f	foot	40	40
53	2010-01-06	13:45	1	194a	2	1	f	auto	75	50
55	2010-01-06	10:00	1	441c	1	1	f	foot	50	50
56	2010-01-06	9:00	1	475c	2	2	m	auto	150	100
57	2010-01-07	11:30	1	Pasiecznik	2	2	m	auto	60	50
58	2010-01-07	8:15	1	526a	2	1	m	foot	50	50
60	2010-01-07	13:30	1	211c	2	1	f	auto	75	75
62	2010-01-07	10:30	1	697a	2	1	f	foot	50	50
63	2010-01-08	10:30	1	247c	2	2	f	auto	200	75
64	2010-01-08	9:45	1	391c	1	1	f	auto	100	100
65	2010-01-08	8:30	1	wieża/Ceg	3	2	m	auto	50	50
69	2010-01-09	14:15	1	211c	2	1	f	auto	75	75
73	2010-01-09	12:45	1	422a	1	2	m	foot	40	20
75	2010-01-11	11:30	1	awiny Gró	2	2	m	foot	250	100
77	2010-01-12	14:30	1	Krynki	3	1	f	foot	75	75
78	2010-01-12	14:30	1	Krynki	3	2	f	foot	150	150
79	2010-01-12	13:30	1	Krynki	3	2	f	foot	250	150
80	2010-01-12	11:45	1	P. Knysz.	3	1	m	foot	75	75
82	2010-01-13	9:30	1	697a	2	1	f	auto	100	100
83	2010-01-15	12:00	1	247c	2	2	f	auto	250	75
86	2010-01-15	13:30	1	130b	2	2	m	foot	100	100

87	2010-01-15	13:00	1	103b	2	2	m	foot	250	200
88	2010-01-15	11:15	1	211c	2	1	f	auto	50	50
89	2010-01-15	10:30	1	441a	1	1	m	foot	100	50
96	2010-01-22	12:15	1	Zabrody	3	2	f	auto	150	150
97	2010-01-22	12:30	1	Zabrody	3	2	f	foot	100	100
98	2010-01-22	12:45	1	Zabrody	3	2	f	auto	50	50
99	2010-01-22	13:30	1	Łozowe	3	2	f	auto	75	75
103	2010-01-22	14:15	1	210c	2	1	m	auto	150	30
104	2010-01-22	11:45	1	Leśna	3	2	f	auto	25	25
105	2010-01-22	10:30	1	Dubiny	3	2	f	foot	25	25
106	2010-01-25	12:30	1	Zabrody	3	2	f	auto	200	200
109	2010-01-26	13:30	1	Czyżyki	3	1	f	foot	100	75
111	2010-01-29	14:15	1	312a	2	2	m	foot	150	150
112	2010-01-29	11:15	1	697a	2	1	f	auto	100	100
113	2010-02-01	14:30	1	697a	2	1	f	foot	80	30
114	2010-02-01	12:15	1	211c	2	1	f	foot	50	50
115	2010-02-04	10:45	1	421b	1	1	f	foot	25	25
117	2010-02-05	11:15	1	Zabrody	3	1	f	foot	50	50
119	2010-02-05	12:00	1	85c	2	1	m	foot	25	25
120	2010-02-08	13:00	1	Zabrody	3	1	f	foot	60	50
121	2010-02-08	13:15	1	Zabrody	3	2	f	auto	125	125
122	2010-02-08	13:30	1	Zabrody	3	2	f	foot	50	50
126	2010-02-15	9:45	1	421b	1	1	f	foot	50	15
127	2010-02-15	10:15	1	391c	1	1	f	auto	100	25
128	2010-02-15	11:15	1	Dubiny	3	2	f	auto	75	50
129	2010-02-15	11:45	1	211c	2	1	f	auto	100	100
130	2010-02-15	12:45	1	Zabrody	3	2	f	foot	100	100
131	2010-02-15	13:45	1	193b	2	1	f	auto	50	50
136	2010-02-19	10:00	1	193b	2	2	f	foot	50	50
138	2010-02-22	11:00	1	echy Orlań	2	1	m	foot	100	10
139	2010-02-22	14:45	1	242c	2	1	m	foot	50	5
140	2010-02-27	11:00	1	Osowe	3	2	m	foot	200	150
141	2010-03-01	10:30	1	391c	1	1	f	foot	125	60
144	2010-03-04	10:30	1	Łozowe	3	2	m	foot	70	70
144	2010-03-04	10:30	1	Łozowe	3	2	m	auto	100	70
145	2010-03-04	13:00	1	echy Orlań	2	1	m	foot	200	200
146	2010-03-05	8:45	1	251d	2	1	m	foot	50	50
146	2010-03-05	8:45	1	251d	2	1	m	auto	100	50
147	2010-03-05	13:15	1	697a	2	1	f	auto	100	100
148	2010-03-05	14:30	1	echy Orlań	2	1	m	foot	50	15
156	2010-03-23	15:00	1	489b	2	1	f	auto	100	40
157	2010-03-26	9:15	1	181c/213a	2	2	f	foot	125	75
158	2010-03-29	13:00	1	697c/729a	2	1	m	auto	350	200
159	2010-03-31	9:00	1	391a	1	1	m	foot	180	130
160	2010-04-02	9:00	2	188c/220b	1	1	f	auto	200	100
161	2010-04-14	16:00	2	248c	1	2	m	auto	200	150
162	2010-04-14	16:45	2	396d	1	1	m	auto	60	40
163	2010-04-20	13:30	2	18c	2	1	f	foot	50	50
166	2010-04-23	11:00	2	339c	2	1	m	foot	60	60
167	2010-04-23	12:00	2	307a	1	1	m	foot	45	45
168	2010-04-26	13:00	2	Długi Bród	2	1	m	foot	40	30
169	2010-04-26	13:00	2	Długi Bród	2	2	m	foot	70	50
170	2010-04-29	12:00	2	601b	2	2	m	foot	40	40

171	2010-04-30	9:45	2	368d	1	1	m	foot	40	40
172	2010-04-30	11:00	2	253d	2	1	f	foot	100	50
173	2010-05-02	14:30	2	Wiluki	2	1	m	foot	50	50
174	2010-05-06	14:45	2	338c	2	1	m	foot	120	100
175	2010-05-17	10:00	2	395a/395b	1	1	m	auto	180	120
176	2010-05-17	10:00	2	395a/395b	1	2	m	auto	40	40
178	2010-06-21	13:30	2	Werstok	2	2	m	foot	25	25
179	2010-06-30	13:00	2	229d	2	1	m	foot	75	50
180	2010-07-02	11:15	2	Werstok	2	2	m	auto	100	50
181	2010-07-02	11:45	2	Jodłówka	2	1	m	foot	80	40
182	2010-07-02	12:45	2	629c	2	1	f	foot	60	30
183	2010-07-06	9:30	2	629a	2	2	f	foot	150	100
184	2010-07-06	10:30	2	sieczniki M	2	1	m	foot	150	75
185	2010-07-07	12:30	2	249c	1	1	f	foot	200	100
186	2010-07-07	14:00	2	98d	1	1	m	foot	50	20
188	2010-07-08	12:45	2	Janowo	2	1	m	foot	70	30
189	2010-07-08	14:30	2	Zabrody	3	2	f	foot	250	200
190	2010-07-08	10:00	2	123b	1	1	m	foot	150	100
193	2010-02-06	10:30	1	Osowe	3	2	m	foot	250	160
194	2010-05-27	15:00	2	397	1	1	m	foot	100	100
195	2010-07-04	8:15	2	279a	1	1	f	foot	80	80
196	2010-01-31	13:00	1	Czyżyki	3	2	f	foot	80	80
197	2010-01-31	10:00	1	281d	3	2	m	auto	200	200
198	2010-01-15	14:00	1	475	2	2	m	auto	150	60
199	2010-01-16	9:30	1	338b	2	2	m	auto	120	50
200	2009-12-15	10:00	1	sieczniki M	2	1	m	foot	50	50
201	2010-01-28	10:00	1	391	1	1	f	foot	100	80
202	2010-01-28	13:30	1	422	1	1	f	foot	100	50
203	2010-01-28	10:00	1	Czyżyki	3	2	f	foot	120	120
204	2010-01-31	12:00	1	Zabrody	3	2	f	foot	150	100
206	2010-06-19	15:30	2	101	2	1	m	foot	20	10
208	2010-06-18	18:00	2	364	1	1	m	foot	10	10
209	2009-12-01	-	1	Białowieża	3	2	m	auto	40	20
213	2009-11-15	6:30	2	Białowieża	2	2	m	foot	40	20
214	2009-11-25	7:00	2	Białowieża	2	2	m	foot	25	25
221	2010-10-01	13:00	2	188a	1	1	f	foot	100	100
224	2010-10-09	17:00	2	395	1	2	f	foot	100	80
225	2010-10-09	13:00	2	101b	2	1	m	foot	30	30
226	2010-10-15	15:00	2	247b	1	1	f	foot	100	95
227	2010-10-11	12:30	2	Biała Straż	3	1	f	auto	120	120
228	2010-10-11	12:45	2	Biała Straż	3	1	f	auto	80	80
229	2010-10-11	14:15	2	Zabrody	3	2	f	foot	200	80
230	2010-10-13	14:15	2	52B	2	1	m	foot	75	75
231	2010-10-15	15:45	2	100a	1	1	m	foot	40	35
233	2010-08-18	5:15	2	526a	1	2	f	auto	70	70
234	2010-09-03	11:15	2	124d	1	2	f	auto	120	120
235	2010-09-22	9:30	2	Wojnówka	3	2	f	auto	140	60
236	2010-09-22	14:00	2	Łozowe	3	2	f	foot	100	70
237	2010-11-08	11:15	2	Biała Straż	3	1	f	foot	75	50
238	2010-11-04	13:45	2	218d	1	1	f	auto	40	40
239	2010-11-04	15:30	2	248d	1	2	f	auto	150	150
240	2010-11-02	11:30	2	Biała Straż	3	2	f	auto	200	120
242	2010-11-08	9:30	2	łowieża W	2	2	m	foot	15	15

243	2010-10-30	12:00	2	bińska Fer	2	2	m	foot	60	40
244	2010-11-09	11:00	2	79c	1	1	m	foot	75	75
245	2010-11-09	12:30	2	Zabrody	3	1	f	foot	150	100
246	2010-11-09	13:30	2	Zabrody	3	2	f	foot	120	120
247	2010-11-09	15:00	2	313d	2	1	m	foot	80	80
250	2010-11-16	10:45	2	725/726	2	1	f	foot	60	60
251	2010-11-16	11:00	2	Górny Gró	2	2	f	foot	120	120
252	2010-11-19	12:00	2	124c	1	1	f	auto	80	80
255	2010-11-26	10:30	2	697a	2	1	f	auto	200	200
256	2010-11-26	12:30	2	Skupowo	3	1	m	foot	60	40
257	2010-11-26	13:15	2	Łozowe	3	2	f	auto	270	180
258	2010-11-27	14:00	2	Podolany	3	1	m	foot	200	200
259	2010-11-30	7:30	2	475a	2	1	m	foot	75	50
260	2010-11-30	7:45	2	422a	1	2	f	auto	80	80
262	2010-11-30	12:00	2	Łozowe	3	2	f	foot	150	100
264	2010-12-03	15:30	1	361d	1	1	m	auto	60	20
265	2010-12-03	14:45	1	211c	2	1	f	foot	80	50
266	2010-12-03	13:30	1	422a	1	1	f	foot	100	70
267	2010-12-11	10:00	1	Trywieża	3	2	f	auto	60	40
268	2010-12-11	11:00	1	Trywieża	3	2	f	auto	100	60
269	2010-12-12	12:00	1	Osowe	3	2	f	foot	150	130
270	2010-12-12	13:00	1	Osowe	3	2	f	foot	120	100
271	2010-12-13	11:45	1	475a	2	2	m	auto	150	100
272	2010-12-13	12:15	1	391c	1	1	f	auto	120	120
273	2010-12-13	13:15	1	248d	2	2	f	auto	200	200
274	2010-12-13	13:30	1	248d	2	2	f	auto	70	70
275	2010-12-13	14:45	1	Zabrody	3	2	f	auto	130	130
277	2010-12-17	10:00	1	211c	2	1	f	auto	100	100
278	2010-12-17	12:15	1	193b	2	1	f	foot	120	100
280	2010-12-17	13:30	1	421b	1	2	m	auto	100	70
281	2010-12-17	14:00	1	475c	2	1	m	foot	60	60
282	2010-12-19	13:15	1	475c	2	2	m	foot	80	80
283	2010-12-19	14:00	1	422a	1	1	f	foot	60	40
284	2010-12-22	8:45	1	312b	2	2	m	auto	100	70
286	2010-12-22	10:45	1	Osowe	3	2	m	auto	200	100
289	2010-12-22	11:00	1	Zabrody	3	2	f	auto	170	100
290	2010-12-22	13:45	1	697a	2	1	f	foot	70	70
293	2011-01-03	15:30	1	Łozowe	3	2	f	foot	200	120
294	2011-01-05	9:15	1	owoberezo	3	2	f	foot	150	100
295	2011-01-05	10:15	1	Kojły	3	2	f	foot	200	150
297	2011-01-06	11:30	1	Czyżyki	3	2	m	foot	200	140
298	2011-01-06	12:30	1	ielszczyzn	3	2	f	foot	150	120
300	2011-02-11	12:00	1	697a	2	1	f	foot	120	90
301	2011-02-10	12:15	1	Łozowe	3	2	f	foot	270	140
303	2011-01-26	9:15	1	Dubiny	3	2	f	foot	270	130
304	2011-01-26	13:15	1	Łozowe	3	2	f	auto	80	80
305	2011-01-26	14:30	1	193a	2	1	f	foot	50	50
308	2011-01-22	11:30	1	391c	1	1	f	foot	100	70
309	2011-03-13	14:30	1	Zabrody	3	1	m	foot	70	40
310	2011-03-13	15:30	1	Zabrody	3	1	m	foot	150	100
311	2011-03-11	14:30	1	441a/c	2	2	m	foot	90	40
312	2011-03-02	10:45	1	Czyżyki	3	2	f	foot	100	50
313	2011-03-01	16:30	1	wieża/Ceg	2	2	m	foot	150	100

314	2011-02-27	13:30	1	393b	1	1	m	foot	200	100
315	2011-02-23	13:00	1	Łozice	3	2	m	auto	200	100
316	2011-02-22	12:00	1	Zabrody	3	2	f	foot	250	150
317	2011-02-21	9:45	1	399b	2	1	m	foot	100	60
320	2011-03-21	13:15	1	Łozowe	3	2	f	auto	200	100
321	2011-03-21	14:30	1	251d	2	1	m	foot	150	100
322	2011-03-18	10:45	1	697a	2	1	f	foot	100	70
323	2011-03-18	9:45	1	419b	1	1	f	foot	150	100
324	2011-03-16	11:15	1	Lipiny	2	1	m	foot	80	40
325	2011-03-16	12:15	1	Zabrody	3	2	f	auto	200	150
326	2011-03-16	12:15	1	Zabrody	3	2	f	foot	150	100
327	2011-03-29	8:00	1	394c	1	1	f	foot	120	80
328	2011-03-29	10:15	1	Lipiny	2	1	m	foot	80	40
329	2011-03-28	13:15	1	Leśna	3	2	f	foot	120	80
335	2011-03-21	12:45	1	Janowo	2	2	m	foot	150	100
336	2011-04-20	9:30	2	248b	2	2	f	foot	50	20
339	2011-04-13	13:30	2	Zabrody	3	2	m	foot	100	70
341	2011-04-11	13:45	2	629c	2	2	f	foot	200	200
343	2011-04-05	14:45	2	62d/63c	2	1	m	auto	150	150
345	2011-05-24	11:30	2	Biała Straż	3	1	m	foot	100	100
346	2011-05-20	10:30	2	282d	2	1	m	foot	100	70
347	2011-05-19	14:15	2	303c	2	2	m	foot	70	50
349	2011-05-06	12:45	2	PB	3	2	f	foot	120	80
352	2011-04-29	13:30	2	423b	1	1	f	foot	80	60
354	2011-08-11	14:00	2	wodziecki	2	1	f	foot	100	100
355	2011-08-11	14:15	2	wodziecki	2	2	m	foot	150	100
356	2011-08-10	10:30	2	374d	2	1	f	foot	100	70
357	2011-08-03	14:30	2	277a	1	1	f	foot	150	100
358	2011-08-03	14:00	2	249c/278a	1	1	f	auto	150	150
359	2011-06-01	14:15	2	PB	3	1	f	auto	70	70
360	2011-05-31	15:30	2	271a	2	1	m	foot	120	120
361	2011-05-31	13:30	2	220c	1	1	f	foot	120	100
362	2011-05-25	13:00	2	276b	1	1	m	foot	100	70
363	2011-05-25	12:15	2	101b	2	1	m	foot	70	50
364	2011-08-24	10:45	2	194b	2	1	f	foot	150	150
365	2011-08-22	11:30	2	Masiewo	2	2	m	foot	200	150
366	2011-08-22	19:45	2	Teremiski	1	2	m	foot	70	50
367	2011-08-18	11:15	2	15b	2	1	f	auto	100	100
368	2011-08-18	13:15	2	578c	1	1	f	foot	100	70
369	2011-08-18	14:15	2	606d	1	1	f	foot	150	100
370	2011-08-17	5:45	2	124d	1	2	f	foot	150	100
371	2011-08-16	12:15	2	Masiewo	2	1	m	foot	70	70
372	2011-08-12	19:00	2	247c	1	2	f	foot	200	100
373	2011-09-12	18:00	2	445d	2	2	m	auto	250	200
374	2011-09-12	17:45	2	421a	1	2	f	auto	100	100
377	2011-09-07	11:30	2	245d	2	1	f	foot	150	100
379	2011-09-02	13:30	2	PB	3	1	f	foot	150	150
381	2011-08-24	13:15	2	Łozowe	3	2	f	foot	300	150
382	2011-10-17	14:15	2	286d	2	1	f	foot	200	100
383	2011-10-13	14:45	2	owiny Gró	2	1	m	auto	75	75
384	2011-10-13	11:15	2	50d	2	1	m	foot	150	100
385	2011-09-28	13:30	2	209d	2	2	m	auto	200	150
386	2011-09-27	16:15	2	609b	1	1	f	foot	50	50

387	2011-09-27	14:45	2	Biała Straż	3	1	f	foot	100	70
388	2011-09-27	12:30	2	241c	2	1	m	foot	100	50
389	2011-09-20	14:15	2	Skryplewo	2	2	m	foot	150	100
391	2011-10-24	12:15	2	400a	2	1	m	foot	100	50
392	2011-10-24	14:15	2	399a/b	2	1	m	foot	50	50
393	2011-10-20	9:30	2	393c	1	1	f	foot	200	150
394	2011-10-20	10:45	2	218d	1	1	f	foot	70	70
396	2011-10-20	14:45	2	bińska Fer	2	2	m	auto	70	50
397	2011-10-20	12:45	2	67c	2	1	m	foot	80	60
399	2011-10-19	14:30	2	275d	1	1	f	foot	200	200
400	2011-11-03	9:45	2	Górny Gród	3	1	f	foot	50	25
401	2011-10-27	14:00	2	312d	2	1	m	auto	50	50
402	2011-10-27	15:30	2	395c	1	1	f	foot	100	50
403	2011-10-27	13:30	2	426a	1	1	m	foot	150	120
405	2011-10-26	7:30	2	Babia Góra	3	2	f	foot	100	90
406	2011-10-26	8:00	2	Babia Góra	3	2	f	auto	300	300
407	2011-10-26	10:30	2	160c	2	1	m	foot	100	50
408	2011-10-25	14:15	2	218c	1	1	f	foot	50	50
409	2011-11-12	6:30	2	wieża/Ceg	2	2	m	foot	80	50
410	2011-11-12	6:45	2	wieża/Ceg	2	2	m	foot	150	80
411	2011-11-12	7:00	2	wieża/Ceg	2	2	m	foot	200	120
412	2011-11-09	10:45	2	339b	2	1	m	foot	150	100
413	2011-11-09	7:45	2	nolany Sac	2	1	m	foot	100	70
414	2011-11-09	7:30	2	Łozowe	1	2	f	foot	300	200
416	2011-11-09	6:20	2	Teremiski	1	2	m	foot	150	100
417	2011-11-04	6:30	2	Teremiski	1	2	m	foot	150	100
418	2011-01-21	13:00	1	Dubiny	2	2	f	foot	120	90
419	2011-01-21	10:45	1	421b	1	2	f	auto	100	30
420	2011-11-15	6:30	2	wieża/Ceg	2	2	m	foot	130	90
421	2011-11-02	11:30	2	470d	2	2	m	auto	180	120
422	2011-11-16	12:00	2	nolany Sac	2	1	m	foot	60	40
423	2011-11-16	12:30	2	Teremiski	1	1	m	auto	45	45
424	2011-11-16	12:45	2	Teremiski	1	2	m	foot	50	40
426	2011-11-18	12:00	2	Wojnówka	3	1	f	foot	70	50
427	2011-10-29	7:00	2	Babia Góra	3	2	f	auto	150	70
428	2011-10-29	8:30	2	160a	2	1	m	foot	50	30
429	2011-12-19	15:00	1	Gruszki	2	2	m	auto	60	30
430	2011-12-20	7:00	1	Gruszki	2	2	m	auto	320	280
431	2011-12-20	7:15	1	Zabrody	3	2	f	auto	270	110
432	2011-12-20	10:45	1	bińska Fer	2	2	f	auto	280	70
433	2011-11-22	14:00	2	owoberezov	2	2	f	foot	280	220
434	2011-11-22	15:15	2	526a	1	2	f	auto	60	60
435	2011-11-30	13:15	2	251d	2	1	m	auto	45	35
436	2011-12-05	10:30	1	393b	1	2	f	auto	70	50
437	2011-12-07	13:00	1	Zabrody	3	2	f	foot	35	35
437	2011-12-07	13:00	1	Zabrody	3	2	f	auto	60	40
438	2011-12-07	14:00	1	67d	2	2	f	auto	65	40
439	2011-12-11	13:15	1	394c	1	1	f	foot	35	35
440	2011-12-13	14:45	1	312b	2	2	m	auto	80	80
441	2011-12-13	15:00	1	312b	2	1	m	foot	90	90
442	2012-01-18	11:00	1	ielszczyzn	2	2	f	foot	120	120
444	2012-01-18	9:15	1	441c	2	2	f	foot	80	40
445	2012-01-18	14:00	1	251d	2	1	m	auto	70	30

446	2012-01-18	14:30	1	475c	2	2	m	auto	120	30
447	2012-01-19	10:15	1	Czyżyki	2	2	f	auto	270	70
448	2012-01-20	12:00	1	Czyżyki	2	2	f	auto	250	80
450	2012-01-20	15:30	1	251d	2	1	m	auto	130	90
451	2012-01-24	9:00	1	397a	1	1	m	foot	80	45
453	2012-01-24	14:30	1	Czyżyki	2	2	f	foot	250	150
454	2012-01-20	15:00	1	193b	2	2	f	foot	100	70
456	2012-01-30	8:00	1	441c	2	2	f	foot	180	110
457	2012-01-30	9:00	1	697a	2	1	f	auto	220	220
459	2012-02-05	13:15	1	475c	2	2	m	auto	80	25
460	2012-02-05	13:45	1	421b	1	1	f	auto	100	20
461	2012-02-05	14:30	1	lowokornin	3	2	m	foot	80	40
462	2012-02-05	14:45	1	lowokornin	3	2	f	auto	300	60
463	2012-02-08	12:45	1	421b	1	2	f	auto	150	10
464	2012-02-08	13:30	1	Puciska	3	2	m	auto	230	30
465	2012-02-08	13:15	1	445c	2	2	m	auto	180	60
466	2012-02-08	13:15	1	445c	2	2	f	auto	190	90
467	2012-02-10	13:30	1	Puciska	3	2	m	auto	200	70
468	2012-02-10	14:30	1	697a	2	1	f	auto	120	80
469	2012-02-09	15:30	1	wieża/Ceg	2	2	m	foot	130	80
471	2012-02-18	13:30	1	421b	1	2	f	foot	125	100
473	2012-02-20	9:30	1	397a	1	1	m	foot	100	40
474	2012-02-20	14:15	1	251d	2	1	m	auto	120	70
475	2012-02-20	14:15	1	251d	2	1	m	foot	80	50
476	2012-02-22	14:00	1	313a	2	2	m	auto	110	60
478	2009-12-23	10:45	1	Lipiny	2	1	m	auto	75	75
479	2010-02-01	11:30	1	470d	2	2	f	auto	100	100
480	2010-07-12	10:00	2	241d	2	1	m	foot	60	30
481	2010-07-13	9:00	2	99c	2	1	f	foot	70	70
482	2010-07-13	9:30	2	78d	2	1	m	foot	60	60
483	2010-07-13	10:00	2	218c	1	1	f	foot	90	90
485	2010-07-14	13:15	2	123b	1	1	f	foot	100	100
486	2010-07-15	14:15	2	526a	1	2	m	auto	70	50
487	2010-07-15	14:30	2	545b	1	1	f	auto	80	60
488	2010-07-19	10:30	2	241d	2	1	m	foot	75	40
489	2010-07-19	12:00	2	Werstok	2	2	m	auto	20	20
490	2010-07-19	13:30	2	660c	1	1	f	foot	40	30
492	2010-07-21	6:15	2	606b	1	1	f	foot	25	25
493	2010-07-21	7:30	2	sieczniki M	2	1	m	foot	50	15
494	2010-07-21	7:30	2	sieczniki M	2	1	m	auto	10	10
495	2010-07-28	12:15	2	248d	1	1	f	foot	70	70
496	2010-08-02	5:45	2	124d	1	1	f	foot	75	75
496	2010-08-02	5:45	2	124d	1	2	f	auto	150	150
497	2010-08-02	14:30	2	246a	1	1	f	foot	130	80
498	2010-08-05	11:45	2	245d	1	2	m	auto	130	130
499	2010-08-09	14:15	2	661a	2	1	f	foot	50	50
500	2010-08-11	14:15	2	210d	2	1	f	foot	60	60
501	2010-08-13	11:45	2	40d	2	1	m	foot	80	80
502	2010-01-16	13:00	1	399d	2	1	m	foot	100	50
504	2009-11-02	11:30	2	470d	2	2	m	auto	200	100
506	2010-08-04	10:00	2	214	2	2	f	foot	100	60
507	2010-08-04	7:30	2	185c	1	1	f	foot	100	80
508	2010-08-04	8:00	2	183a	1	1	m	auto	20	20

510	2010-08-20	9:00	2	124d	1	1	f	foot	150	120
511	2012-03-15	14:15	1	Lipiny	2	1	m	foot	65	45
513	2012-03-15	15:15	1	251c	2	1	m	foot	90	60
514	2012-03-15	12:00	1	396b	1	1	m	foot	90	70
520	2012-03-16	15:15	1	193b	2	1	f	foot	80	70
520	2012-03-16	15:15	1	193b	2	1	f	auto	90	90
521	2012-03-22	6:45	1	Podolany	2	2	m	auto	250	175
522	2012-03-22	6:45	1	Podolany	2	2	m	foot	120	80
523	2012-03-22	8:45	1	697A	2	1	f	auto	175	175
524	2012-03-23	9:45	1	Lipiny	2	1	m	foot	70	60
526	2012-03-23	18:45	1	441C	2	2	m	auto	280	230
528	2012-03-30	15:30	1	Teremiski	1	2	m	auto	70	20
529	2012-04-01	18:30	2	Teremiski	1	2	m	foot	150	110
530	2012-04-05	13:45	2	Łozowe	3	2	f	auto	200	150
531	2012-04-05	12:00	2	Lipiny	2	1	m	foot	100	40
533	2012-04-05	9:15	2	393B	1	1	f	foot	120	70
534	2012-04-11	17:45	2	248D	1	1	f	auto	110	80
538	2012-04-27	7:15	2	365D	1	1	f	foot	70	50
539	2012-04-25	12:30	2	39A	3	2	f	auto	90	70
540	2012-04-23	14:15	2	Gruszki	2	2	m	auto	100	40
541	2012-04-23	13:45	2	220C	1	1	f	foot	90	90
543	2012-04-20	11:30	2	364B	1	1	m	foot	70	50
544	2012-05-09	13:45	2	215D	1	1	f	auto	130	110
545	2012-05-09	13:45	2	215D	1	1	f	foot	70	60
546	2012-05-14	12:30	2	155A	1	1	f	foot	140	140
547	2012-05-25	14:00	2	187B	1	2	f	foot	150	110
549	2012-06-28	16:15	2	124B	1	1	f	foot	110	70
550	2012-05-24	15:45	2	Teremiski	1	2	m	foot	170	120
552	2012-07-14	13:00	2	124B	1	1	f	foot	40	30
553	2012-07-15	16:45	2	124A	1	1	f	foot	30	20
554	2012-07-15	17:15	2	99D	1	1	f	foot	30	20
556	2012-07-20	6:30	2	124D	1	2	f	foot	90	60
557	2012-07-20	7:30	2	Łańczyno	2	2	f	auto	150	150
558	2012-07-20	7:30	2	Łańczyno	2	2	f	foot	80	60
560	2012-08-22	12:45	2	276A	1	1	f	auto	125	70
562	2012-10-04	15:00	2	248D	1	2	f	foot	150	120
563	2012-09-27	7:30	2	102A	2	1	m	auto	80	80
564	2012-10-13	7:00	2	wieża/Ceg	2	2	m	foot	120	70
565	2012-10-12	15:15	2	423B	1	1	f	foot	150	150
565	2012-10-12	15:15	2	423B	1	1	f	auto	170	170
566	2012-10-12	16:00	2	278D	1	1	m	auto	160	160
567	2012-10-12	16:45	2	Budy	1	2	m	foot	25	15
568	2012-10-14	17:15	2	Budy	1	2	m	foot	150	20
570	2012-10-25	11:00	2	422B	1	1	m	foot	60	40
571	2012-10-28	15:30	2	211C	2	1	m	auto	100	80
573	2012-10-26	12:30	2	Łozowe	3	2	f	foot	250	90
574	2012-10-29	18:00	2	eża, ul. Sp	2	2	m	foot	50	50
577	2012-11-05	12:30	2	697C	2	1	f	foot	75	40
578	2012-11-05	13:15	2	664A	2	1	f	foot	75	60
579	2012-07-25	9:00	2	124D	1	1	f	foot	70	70
580	2012-07-30	9:15	2	527B	1	1	f	foot	50	50
581	2012-07-11	11:30	2	Grudki	2	1	m	foot	75	65
583	2012-07-16	11:30	2	154A	1	1	f	foot	85	85

586	2012-07-20	11:30	2	154A	1	1	f	foot	80	80
587	2012-07-22	14:15	2	Grudki	2	1	m	foot	80	80
588	2012-07-23	11:45	2	124D	1	2	f	foot	100	100
589	2012-07-25	10:30	2	124B	1	1	f	foot	100	100
591	2012-08-01	10:30	2	154B	1	1	f	foot	80	80
592	2010-07-21	13:30	2	579D	1	1	f	foot	50	50
593	2010-07-28	12:15	2	544C	1	1	m	foot	60	60
594	2010-07-31	19:00	2	549B	1	1	f	foot	80	80
595	2010-08-04	14:00	2	578B	1	1	f	foot	50	50
596	2010-08-08	13:00	2	582A/549C	1	1	m	foot	40	40
597	2010-08-11	13:00	2	642C	1	1	f	foot	20	20
598	2010-08-12	13:45	2	549C	1	1	f	foot	35	35
599	2010-08-23	15:30	2	525D/549E	1	2	m	foot	55	55
600	2010-08-24	10:00	2	544C	1	1	f	foot	55	55
601	2010-08-09	11:00	2	288B	2	1	m	foot	70	70
602	2010-08-10	11:15	2	316D	2	1	m	foot	50	50
603	2010-08-12		2	316B	2	1	m	foot	45	45
604	2010-08-25	12:45	2	316B	2	1	f	foot	60	60
606	2012-11-11	13:00	2	Teremiski	1	2	m	foot	40	25
607	2012-11-11	15:30	2	244C/D	2	2	f	auto	90	80
608	2012-11-18	14:15	2	475C/D	2	1	m	foot	80	30
610	2012-12-01	14:45	1	na Białowi	2	1	m	foot	120	70
611	2012-12-02	14:00	1	na Białowi	2	1	m	foot	80	40
612	2013-01-12	9:00	1	475A/B	2	2	m	auto	60	40
613	2013-01-12	10:00	1	Zabrody	3	2	f	foot	100	70
614	2013-01-12	13:00	1	391C	1	1	f	auto	120	25
615	2013-01-12	13:00	1	391C	1	1	f	auto	10	10
616	2013-01-12	8:30	1	422B	1	1	f	auto	40	40
617	2012-12-29	13:00	1	475C	2	2	m	auto	110	110
618	2013-01-07	11:00	1	475B	2	2	m	auto	75	75
619	2013-01-11	14:15	1	391C	1	1	f	auto	170	120
625	2013-01-18	10:00	1	697C	2	1	f	auto	130	130
626	2013-01-18	10:00	1	697C	2	1	f	foot	160	100
628	2013-01-18	13:15	1	422B	1	1	f	foot	70	40
630	2013-01-20	16:00	1	475B	2	2	m	foot	75	50
631	2013-01-21	14:15	1	475A	2	2	m	auto	110	70
632	2013-01-21	14:15	1	475A	2	1	m	foot	80	50
633	2013-01-25	10:00	1	312/313	2	2	m	auto	130	100
635	2013-01-25	14:30	1	Kotówka	3	2	f	foot	200	120
636	2013-01-25	15:30	1	211C	2	1	f	auto	130	130
640	2013-02-25	15:00	1	475B	2	2	m	auto	70	30
641	2013-02-28	11:30	1	496D/524E	2	1	m	auto	60	20
